# DDX50 Is a Viral Restriction Factor That Enhances IRF3 Activation

**DOI:** 10.3390/v14020316

**Published:** 2022-02-03

**Authors:** Mitchell A. Pallett, Yongxu Lu, Geoffrey L. Smith

**Affiliations:** 1 Department of Pathology, University of Cambridge, Tennis Court Road, Cambridge CB2 1QP, UK; mitchell.pallett10@imperial.ac.uk (M.A.P.); yl581@cam.ac.uk (Y.L.); 2 MRC Centre for Molecular Bacteriology and Infection, Department of Infectious Disease, Imperial College London, London SW7 2AZ, UK

**Keywords:** IRF3, antiviral, DExD-box helicase, RLR, Zika, HSV, VACV

## Abstract

The transcription factors IRF3 and NF-κB are crucial in innate immune signalling in response to many viral and bacterial pathogens. However, mechanisms leading to their activation remain incompletely understood. Viral RNA can be detected by RLR receptors, such as RIG-I and MDA5, and the dsRNA receptor TLR3. Alternatively, the DExD-Box RNA helicases DDX1-DDX21-DHX36 activate IRF3/NF-κB in a TRIF-dependent manner independent of RIG-I, MDA5, or TLR3. Here, we describe DDX50, which shares 55.6% amino acid identity with DDX21, as a non-redundant factor that promotes activation of the IRF3 signalling pathway following its stimulation with viral RNA or infection with RNA and DNA viruses. Deletion of DDX50 in mouse and human cells impaired IRF3 phosphorylation and IRF3-dependent endogenous gene expression and cytokine/chemokine production in response to cytoplasmic dsRNA (polyIC transfection), and infection by RNA and DNA viruses. Mechanistically, whilst DDX50 co-immunoprecipitated TRIF, it acted independently to the previously described TRIF-dependent RNA sensor DDX1. Indeed, shRNA-mediated depletion of DDX1 showed DDX1 was dispensable for signalling in response to RNA virus infection. Importantly, loss of DDX50 resulted in a significant increase in replication and dissemination of virus following infection with vaccinia virus, herpes simplex virus, or Zika virus, highlighting its important role as a broad-ranging viral restriction factor.

## 1. Introduction

Interferon (IFN) regulatory factor 3 (IRF3)-dependent signalling is crucial for pathogen clearance and host survival in response to infection by many viral and bacterial pathogens [1,2]. IRF3 signalling is tightly regulated and is triggered by intracellular cytoplasmic/endoplasmic detection of viral RNA (dsRNA/5′-ppp/pp-RNA) [3] and DNA by pattern recognition receptors (PRRs) [4]. The retinoic acid-inducible gene I (RIG-I)-like receptors (RLRs) DDX58 (RIG-I), MDA5, and LPG2; the DExD/H-box helicases DDX21, DDX1, DHX36, DDX60, DDX3, DHX9, DHX29, and DHX33; and the Toll-like receptor (TLR)-3 bind directly to, or form complexes with, viral dsRNA or 5′-ppp-RNA to activate downstream kinases and induce expression of viral restriction factors [5,6]. In brief, RIG-I/MDA5 activation leads to mitochondrial antiviral signalling protein (MAVS)-dependent autophosphorylation of TANK-binding protein-1 (TBK1). In turn, TBK1 phosphorylates IRF3, leading to its dimerisation and translocation into the nucleus. In parallel, phosphorylation of IκKβ leads to the phosphorylation and degradation of IκBα and the consequential release and translocation into the nucleus of nuclear factor kappa-light-chain-enhancer of activated B cells (NF-κB) [7]. IRF3 and NF-κB transcriptionally upregulate the expression of IFNs, inflammatory cytokines, and chemokines, including IFNβ and C-X-C motif chemokine 10 (CXCL10/IP-10), and IRF3-dependent viral restriction factors [8,9]. These events establish the host antiviral innate immune response, restricting viral replication and aiding clearance of infection.

The prototypical RLR, RIG-I, comprises a DExD-box ATPase-dependent RNA helicase with an *N*-terminal caspase activation and recruitment domain (CARD) and a C-terminal auto-inhibitory regulatory domain (RD). Under resting conditions, RIG-I is held in an autoinhibitory conformation. Upon agonist (5′-ppp/pp-RNA or short dsRNA) binding to the RD, RIG-I undergoes conformational change, tetramerization, and activation [10]. K63-linked ubiquitylation of RNA-bound RIG-I oligomers through interaction with the co-receptor E3 ligase RIPLET induces higher-order clustering of RIG-I. This in turn promotes and facilitates signal transduction via complex formation with the adaptor MAVS [11,12]. Similarly, MDA5 signalling converges at MAVS activation but differs from RIG-I receptor signalling due to alterations in ligand specificity. Additionally, TLR3 differs in cellular localisation and signals in a TIR domain-containing adapter molecule 1 (TICAM-1 or TRIF) and TBK-dependent manner but independent of MAVS. MDA5 recognises high molecular weight dsRNA [13] or mRNA lacking 2′-*O*-methylation at the 5′ cap [14], whereas TLR3 detects dsRNA in the endosomal compartment or extracellular milieu. These subtle differences mean that during infection, numerous RLRs and RNA sensors are activated in parallel or independent of one another and this is dependent upon the cell type, pathogen, and/or specific ligands present.

Other DExD/H-Box RNA helicase family members act either in concert with RIG-I, as independent RNA sensing complexes, or as components of the signal transduction pathway to contribute to the activation of IRF3 signalling in response to viral PAMPs. These include DDX60 [15,16], DDX1, DHX36, DDX21 [17], DHX33 [18], DDX3 [19], and DHX29 [6]. Miyashita and colleagues identified DDX60 as a component of RLR-dependent signalling, acting through RIG-I and MDA5 to trigger optimal IRF3-dependent gene expression [15,16]. Alternatively, DDX1, DHX36, and DDX21 form a cytoplasmic complex with TRIF upon detection of 5′-ppp-RNA or dsRNA (PolyIC). DDX1 and DHX36 interaction, and TRIF recruitment, are DDX21 dependent, whereas DDX1 acts as the complex RNA sensor. Interestingly, this complex acts independently of TLR3, RIG-I, and MDA5 in mouse dendritic cells (DCs) and mouse embryonic fibroblasts (MEFs) [17]. Aside from RIG-I, the role of DExD-Box RNA helicases in antiviral signalling can be highly context specific, with different roles based on the cell types or viruses used [20,21,22].

A recent RNAi screen implicated the relatively uncharacterised DExD-Box RNA helicase proteins DDX17 and DDX50 as putative positive regulators of *IFNβ* promoter activity in response to cytoplasmic 5′-ppp-RNA [23]. DDX50 is a paralogue of DDX21, sharing 55.6% amino acid identity [24]. DDX21 (Guα; nucleolar protein 2) and DDX50 (Guβ; nucleolar protein 1) are the only members of the Gu family of nucleolar RNA helicases and contain a highly homologous GUCT (Gu C-terminal) domain, which is followed by an arginine-serine-rich C-terminal tail in DDX50 [25]. DDX50, as the name suggests, is localised to the nucleoli and in vitro assays have demonstrated that both DDX21 and DDX50 have ATPase and helicase activity; however, DDX50 lacks RNA folding activity [25]. Although DDX21 and DDX50 may have arisen by gene duplication on chromosome 10, these proteins have non-redundant roles. DDX21 targets RNA substrates with a 21- or 34-nt duplex and 5′-overhangs, whereas DDX50 targets only 21-nt duplex RNA for unwinding [25]. On the other hand, DDX50 is required for optimal DDX21 unwinding activity, suggesting some co-dependence [25]. Little is known about the biological function of DDX50, with one study suggesting it may be involved in MAP-kinase signalling through interaction with c-Jun [26]. By using CRISPR technology, we knocked out *Ddx50* in MEFs and *DDX50* in human embryonic kidney 293T cells (HEK293Ts) and found enhanced IRF3-dependent gene transcription and cytokine synthesis and secretion in response to cytoplasmic dsRNA, and RNA or DNA virus infection. Further, DDX50 functions independently of the RNA sensor DDX1 and acts upstream of IRF3 phosphorylation. Significantly, we demonstrated a role for DDX50 as a restriction factor for the DNA viruses vaccinia virus (VACV) and herpes simplex virus type 1 (HSV-1), and the RNA virus Zika (ZIKV).

## 2. Materials and Methods

### 2.1. Cells, Plasmids, Reagents, and Viruses

All reagents were purchased from Sigma unless stated otherwise. BS-C-1 (ATCC CCL-26), U2OS (ATCC HTB-96), HEK293T (ATCC CRL-11268), and immortalised mouse embryonic fibroblasts (MEFs) were all grown in Dulbecco’s modified Eagle’s medium (DMEM) high glucose (Gibco, ThermoFisher Scientific, Waltham, MA, USA), supplemented with 10% foetal bovine serum (FBS; Pan Biotech), 50 µg/mL penicillin/streptomycin (P/S), non-essential amino acids (NEAA). HeLa (ATCC CCL-2), and human fibroblasts (HFs) clone EF-1-F (sourced from Doorbar lab, University of Cambridge, Cambridge, UK) were grown in MEM (Gibco, ThermoFisher Scientific, Waltham, MA, USA) supplemented with 10% FBS, 50 µg/mL P/S, and non-essential amino acids (NEAAs). All cells were grown at 37 °C in a 5% CO_2_ atmosphere and were routinely screened for mycoplasma contamination. All plasmids constructed in this study are listed in Table S1. Vaccinia virus (VACV) strain Western Reserve (WR) recombinant vA5-GFP [27], modified vaccinia virus Ankara (MVA) [28], HSV-1 S17 GFP-Vp26 [29], and HSV-1 ΔICP0 [30] were described previously. The titre of infectious viral particles (plaque-forming units per mL, p.f.u./mL) was determined by plaque assay on BS-C-1 cells for VACV WR and on U2OS for HSV-1. Sendai virus Cantell strain (Licence No. ITIMP17.0612A) at 4000 haemagglutining units per mL (HAU/mL) was a gift from Steve Goodbourn, St George’s Hospital Medical School, University of London, London, UK. ZIKV engineered to express a mCherry marker [31] was a kind gift from Dr. Trevor Sweeney, Department of Pathology, University of Cambridge.

### 2.2. CRISPR-Cas9 Generation of Knockout Cell Lines

Guide RNA design and synthesis, and pX459 plasmid construction was performed following the Zhang lab protocol [32]. Specific guide RNAs are described in Table S1. To generate KOs, MEFs were transfected with pX459 plasmids using LT1 following the manufacturer’s protocol. Then, 24 h post transfection, MEFs and HEK293Ts were treated with 4 and 1 µg/mL puromycin (Invitrogen, ThermoFisher Scientific, Waltham, MA, USA) for 48 h, respectively. Single cell clones were selected by limiting dilution, expanded, and screened for DDX50 protein levels by immunoblot. To confirm successful knockouts, the genomic DNA of selected clones was purified following the manufacturer’s protocol (Qiagen, Germantown, MD, USA; QIAamp DNA mini kit). *Ddx50* was amplified using the primer pair gagcgtccttcctggagattg/ctcaagtctgcccatctctcg and *DDX50* was amplified using the primer pair ctgtgtcaccaggtggcatg/gactcgtgtaactttctttccc. Single allele PCR amplicons were then cloned into pCR2.1-TOPO by blunt end ligation (Thermofisher Scientific, Waltham, MA, USA) and 10 clones were sequenced for each KO cell line clone. Single allele sequencing results were compared to the sequence results of the genomic DNA PCR amplicon to check all alleles had been identified and that all mutations resulted in frameshift truncations.

### 2.3. pLDT and pCW57 Cell Line Generation

WT and *Ddx50^−/−^* MEF and WT HF cell lines inducibly overexpressing DDX50 were obtained by transduction using lentivirus vectors. pLDT and pCW57 cell lines were generated as described [33] with the following alterations. MEFs and HFs were selected in 4 µg/mL puromycin (Invitrogen, ThermoFisher Scientific, Waltham, MA, USA), followed by single cell selection. For HFs, pLDT-TetR-GFP was co-packaged along with the pLDT-MCS plasmids and selected for with 500 µg/mL neomycin (Gibco, ThermoFisher Scientific, Waltham, MA, USA).

### 2.4. Luciferase Reporter Assay

HEK293T, HF, and MEF cell lines were transfected with 10 ng of the internal control plasmid pTK-Renilla (pRL-TK; Promega, Southampton, UK) or 60 ng of the reporter plasmid pLuc-NF-κB (R. Hofmeister, University of Regensburg, Regensburg, Germany) or pLuc-IFNβ (T. Taniguchi, University of Tokyo, Japan) using LT1 transfection reagent and following the manufacturer’s instructions (MirusBio Ltd., Madison, WI, USA). Where stated, plasmids encoding TRIF, MAVS, or TBK-1 (K.A. Fitzgerald, University of Massachusetts Medical School, USA) were co-transfected. Then, 24 h post-transfection, cells were stimulated with IL-1α (Invivogen, Toulouse, France) or TNFα (Invivogen, Toulouse, France) at 100 ng/mL or transfected with 5 µg/mL high molecular weight (HMW) PolyIC (Invivogen) using Liopfectamine 2000 (Invitrogen, ThermoFisher Scientific, Waltham, MA, USA), or mock-transfected with lipofectamine only, or treated exogenously with 5 µg/mL PolyIC, or left unstimulated for 6 h in DMEM or MEM with 2% FBS. Alternatively, cells were stimulated by SeV infection at 40 HAU/mL for 24 h. Following stimulation, cells were lysed in 1× Passive lysis buffer (Promega, Southampton, UK) and Firefly luciferase and Renilla luminescence were measured using the MARS data analysis software on the FLUOstar Omega Luminometer (BMG Labtech, Aylesbury, UK). Relative luminescence levels were calculated by normalising Firefly luminescence to Renilla and data are presented relative to the non-stimulated untreated condition, or EV where relevant, for each cell line. Each condition was performed with quadruplicate technical replicates and is representative of two biological repeats. WT and *Ddx50^−/−^* MEF and WT HF cell lines inducibly overexpressing DDX50 were obtained by transduction using lentivirus vectors. pLDT and pCW57 cell lines were generated as described [33] with the following alterations. MEFs and HFs were selected in 4 µg/mL puromycin (Invitrogen, ThermoFisher Scientific, Waltham, MA, USA), followed by single cell selection. For HFs, pLDT-TetR-GFP was co-packaged along with the pLDT-MCS plasmids and selected for with 500 µg/mL neomycin (Gibco, ThermoFisher Scientific, Waltham, MA, USA).

### 2.5. Retroviral Transduction and Stable Knockdown Cell Lines

pMX-CMV-YFP Micro-RNA30-based (miR-30) gene silencing constructs were generated and transduced as described previously [34] using the primers and plasmids in Table S1. Sequences were as follows: *DDX1* clone 1 TCCGGGCAATCAAGGAACATAA; *DDX1* clone 2 AGATGTGGTCTGAAGCTATTAA and LacZ (non-targeting negative control) ACGTCGTATTACAACGTCGTGA. HEK293T WT or HEK293T *DDX50* KO were transduced and selected with 1 µg/mL puromycin and sorted for high YFP expression using the MoFlo Astrios Cell Sorter (Beckman Coulter Life Sciences, Indianapolis, IN, USA).

### 2.6. ELISAs and RT-qPCR

MEFs were seeded in DMEM with 2% FBS and HEK293Ts were seeded in DMEM with 10% FBS. After 18 h, cells were mock-transfected or transfected with 5 µg/mL HMW PolyIC (Invivogen) using Lipofectamine 2000 (Thermofisher) for 7 h or infected with 40 HAU/mL SeV for 4.5 or 24 h where stated. The culture medium was cleared by centrifugation at 17,000× *g* and stored at −20 °C before analysis by ELISA. The level of human or mouse CXCL10/IP-10 was determined using a DuoSet ELISA kit (R&D Systems, Minneapolis, MN, USA) and the level of mouse IL-6 was determined using a DuoSet ELISA kit (R&D systems) following the manufacturer’s instructions. Data were collected and analysed using the MARS data analysis software on the FLUOstar Omega Luminometer (BMG Labtech, Aylesbury, UK). Experiments were carried out in triplicate and measured with technical repeats, unless stated otherwise. RNA extraction, cDNA synthesis, and RT-qPCR were carried out as described previously using first strand synthesis (Invitrogen) [35]. qPCR was performed using the primers indicated in Table S2.

### 2.7. Immunoprecipitations

HeLa cells were transfected with pLDT-hDDX50-HA and co-transfected with pCDNA3-GFP-Flag or pCDNA3-TRIF-cTAP where stated. For MEFs, DDX50-HA pCW57 cell lines were induced with 2 µg/mL doxycycline 24 h prior to transfection with pCDNA3-GFP-Flag or pCDNA3-TRIF-cTAP. WT and *DDX50^−/−^* HEK293Ts were transfected with pCDNA3-TRIF-cTAP. Then, 24 h post transfection, cells were stimulated by transfection with 5 µg/mL PolyIC or infected with SeV at 20 HAU/mL for 1 h where stated. Following stimulation, cells were washed and lysed in 50 mM Tris pH 7.6, 150 mM NaCl, 1% NP40 (IGEPAL CA-630), 1 mM EDTA, 10% glycerol, and supplemented with protease inhibitor. Proteins were immunoprecipitated as described [36] with M2 Flag-beads or HA-beads. After the final wash, beads were incubated in 4× sample buffer (Tris 0.5 M pH 6.8, 40% glycerol, 6% SDS, 1% bromophenol blue, and 0.8% β-mercaptoethanol), boiled, and analysed by immunoblotting.

### 2.8. Immunoblotting

Samples were prepared by the addition of 4× sample buffer, boiled and separated by gel electrophoresis in Tris-glycine SDS (TGS) buffer (20 mM Tris, 192 mM glycine, 1% (*w*/*v*) SDS), and transferred to a nitrocellulose membrane (GE Healthcare, Chicago, IL, USA) in Tris glycine (TG) buffer (20% methanol, 20 mM Tris-HCl pH 8.3, 150 mM glycine) using the Turboblot system (BioRAD, Hercules, CA, USA). Membranes were blocked in 5% milk in Tris-buffered saline (10 mM Tris, 150 mM NaCl) pH 7.4 with 0.1% (*v*/*v*) Tween-20 (TBS-T) for 1 h before incubating with the primary antibody overnight at 4 °C. Primary antibodies: rabbit monoclonal anti-Flag (F7425), anti-DDX50 (Abcam, Cambridge, UK; ab109515), anti-IRF3 Ser386 (Abcam, ab76493), rabbit polyclonal anti-HA (H6908), mouse monoclonal anti-Flag (F1804), anti-α-tubulin (Millipore; 05-829), anti-DDX50 (Santa Cruz, Dallas, TX, USA; sc-81077), anti-DDX1 (Santa Cruz, Dallas, TX, USA; sc-271438), anti-LaminA/C (Abcam, Cambridge, UK; ab8984), mouse polyclonal anti-IRF-3 S396 (CST, Danvers, MA, USA; #4947S), or mouse monoclonal anti-D8 clone AB1.1 [37]. Membranes were washed 3 times in TBS-T before incubation with secondary antibodies for 1 h. Secondary antibodies were goat anti-rabbit IRDye 800CW (926-68032211; LiCOR, Lincoln, NE, USA), and goat anti-mouse IRDye 608LT (926-68020; LiCOR) or, for immunoprecipitated samples, biotin-anti-mouse light chain followed by streptavidin IRDye 680LT (926-68031; LiCOR, Lincoln, NE, USA) was used. Finally, membranes were washed 3 times in TBS-T, dried, and imaged using the LiCOR system and Odyssey software (LiCOR, Lincoln, NE, USA). For protein level comparisons, densitometry was calculated using ImageJ (National Institutes of Health, Bethesda, MD, USA).

### 2.9. Virus Growth Assays

To measure viral spread, confluent monolayers of WT or KO MEFs (in 6-well plates) were infected with 80 p.f.u. of vA5-GFP or 200 p.f.u. of HSV-1 S17 Vp26-GFP in DMEM with 2% FBS. Alternatively, for the single step virus replication analysis, cells were infected with 5 p.f.u./cell of vA5-GFP. Plates were rocked regularly at 37 °C for 2 h before incubation at 37 °C for the indicated times. Plaques were imaged using an Axiovert.A1 inverted fluorescence microscope connected to a Zeiss MRc color camera (Zeiss, Oberkochen, Germany) and processed using Axiovision Rel. 4.8 imaging software (Zeiss, Oberkochen, Germany). To determine the viral titre, the medium and cells were collected, freeze-thawed 3 times, sonicated at 2.0 for 20 s 3 times (for VACV only), and titrated on BS-C-1 or U2OS cells for VACV and HSV-1, respectively. For ZIKV infection and titration, 3 × 10^6^ parental HEK293T or DDX50^−/−^ cells were seeded on poly-D-lysine pre-coated 6-well plates. Cells were infected with ZIKV at 0.1 p.f.u./cell the next day. Three days p.i., supernatants of the infected cells were collected, and virus infectivity was titrated by plaque assay on Vero E6 cells. To titrate ZIKV samples, Vero E6 cells on 6-well plates (90% confluence) were infected for 2 h, the inoculum was removed, and cells were incubated in MEM with 1.5% carboxymethyl cellulose for 5 days. Cells were then fixed with 4% paraformaldehyde (PFA) and stained with toluidine blue.

### 2.10. Cell Sub-Fractionation

Following stimulation for the times indicated, cells were washed in PBS and fractionated using the NE-PER™ Nuclear and Cytoplasmic Extraction Kit following the manufacturer’s protocol (ThermoFisher).

### 2.11. Immunofluorescence

Briefly, cells were fixed in 4% PFA/PBS for 20 min, washed in PBS, quenched in 150 mM NH_4_Cl/PBS for 10 min, and permeabilised in 0.1% Triton X-100/PBS for 10 min, before a final wash and block in 5% FBS/PBS. Cells were stained by inverted incubation in 5% FBS/PBS with anti-rabbit HA (dilution 1:100) antibody for 1 h, washed in 5% FBS/PBS, and incubated for a further 30 min with the secondary goat anti-rabbit IgG Alexa-Fluor 488 (Jackson immunoresearch, West Grove, PA, USA; 111-545-003). Coverslips were mounted in Mowiol (10% *w*/*v* Mowiol4–88 (CalBiochem, San Diego, CA, USA), 25% *v*/*v* glycerol, 100 mM Tris-HCl pH 8.5, 0.5 µg/mL DAPI (4′,6-diamidino-2-phenylindole, Sigma, Castleford, UK) and images were acquired using a Zeiss LSM780 confocal laser scanning microscopy system and processed using the Zeiss Zen microscope and Axiovision 4.8 software (Zeiss, Oberkochen, Germany).

### 2.12. Statistics

All experiments are presented as technical or biological averages where stated. Data presented are the mean ± SD. Assays comparing two groups were analysed by the two-tailed unpaired *t*-test. The one-way or two-way ANOVA followed by Tukey’s multiple comparison post-hoc test was applied to analyse differences between the means of more than two groups when considering one or two independent variables, respectively. Statistical analysis was performed with GraphPad Prism 9 (GraphPad, San Diego, CA, USA). Exact *p* values are shown to 5 significant figures.

## 3. Results

### 3.1. DDX50 Is a Novel Factor Required for the Innate Immune Response to Nucleic Acid

**Figure 1:**
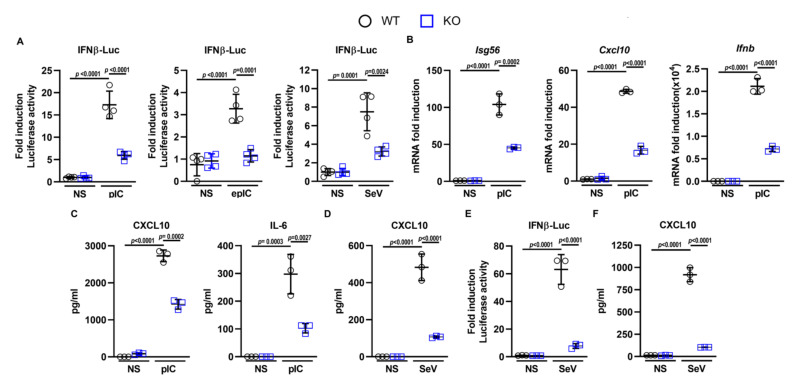
DDX50 (RH-II/Guβ) is required for innate immune signalling in response to intracellular nucleic acid. (**A**) Firefly luciferase activity of WT or *Ddx50^−/−^* MEFs transfected with plasmids encoding Firefly Luciferase under the *Ifnβ* promoter and Renilla. Cells were left untreated or treated with 5 µg/mL extracellular PolyIC (epIC), transfected with 5 µg/mL PolyIC (pIC) for 6 h, or infected with Sendai virus (SeV) for 24 h. (**B**) WT or *Ddx50^−/−^* MEFs were transfected with lipofectamine only or 5 µg/mL pIC for 7 h, and the fold induction of *Isg56*, *Cxcl10*, or *Ifnb* mRNA levels, relative to *Gapdh*, were analysed by RT-qPCR. (**C**) Secreted levels of CXCL10 and IL-6 in the medium at 7 h post transfection with PolyIC or (**D**) 4.5 h post infection with SeV were analysed by ELISA. (**E**) Firefly Luciferase activity of WT or *DDX50^−/−^* HEK293Ts transfected with plasmids encoding Firefly Luciferase under the *Ifnβ* promoter and Renilla under the TK promoter. Cells were infected for 24 h with SeV or left untreated. Data are representative of at least three independent experiments. (**F**) Secreted levels of CXCL10 in the medium at 24 h post infection of WT or *DDX50^−/−^* HEK293Ts with SeV were analysed by ELISA. Data are representative of at least three independent experiments. For all panels, statistical significance was determined by performing a two-way ANOVA test followed by Tukey’s multiple comparison post-hoc.

To investigate the putative role of DDX50 in cytoplasmic RNA sensing, CRISPR-mediated *Ddx50*/*DDX50* knockouts (KOs) were generated in MEFs and HEK293Ts. Successful KO was confirmed by immunoblotting (Figure S1B,E) and genomic sequencing of individual alleles (Figure S1A,C,D,F). Sequencing showed frameshifts in exon 1 (Figure S1C) and exon 4 (Figure S1F) producing nonsense mutations and introduction of an early stop codon. No differences in morphology or growth properties between the wild type (WT) and KO cells were observed (data not shown). Initially, the contribution of DDX50 to IRF3 signalling in response to RLR agonists was investigated. Cells were co-transfected with a Firefly Luciferase reporter plasmid under the control of the *Ifnβ* promoter (pLuc-IFNβ) and an internal control plasmid constitutively expressing Renilla Luciferase (pTK-RL). These cells were further mock-transfected or transfected with PolyIC (dsRNA analogue), infected with an RNA virus (Sendai virus (SeV) or treated with extracellular PolyIC. *Ifnβ* promoter activity was then measured relative to Renilla luminescence and non-stimulated controls. Promoter activity was significantly diminished in KO cells in comparison to WT cells in response to all stimuli (Figure 1A), validating the results observed in the initial RNAi screen [23]. Consistent with this observation, knockout of *Ddx50* reduced the expression of endogenous IRF3-dependent genes (*Isg56*, *Cxcl10*, and *Ifnb*) in response to PolyIC and SeV as measured by RT-qPCR (Figure 1B) and ELISA (CXCL10 and IL-6; Figure 1C,D). Collectively, this indicates that DDX50 affects the IRF3 or IRF3 and NF-κB branch of signalling in response to cytoplasmic dsRNA.

**Figure 2:**
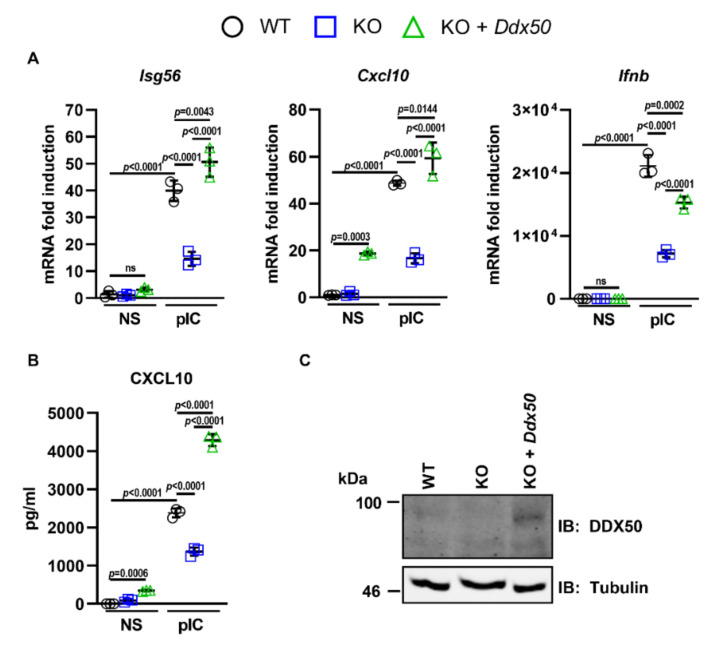
DDX50 rescues nucleic acid-induced signalling in *Ddx50^−/−^* MEFs. (**A**–**C**) WT MEFs transduced with pLDT-EV and *Ddx50^−/−^* MEFs transduced with pLDT-EV or pLDT-*Ddx50* were transfected with lipofectamine only or 5 µg/mL PolyIC for 7 h. (**A**) *Isg56*, *Cxcl10*, or *Ifnb* mRNA levels, relative to *Gapdh*, were analysed by RT-qPCR and (**B**) secreted CXCL10 was measured by ELISA. Representative of at least two independent experiments. For all panels, statistical significance was determined by performing a two-way ANOVA test followed by Tukey’s multiple comparison post-hoc test. ns, non-significant. (**C**) Expression of DDX50 was confirmed by SDS-PAGE and immunoblotting using an anti-DDX50 antibody.

**Figure 3:**
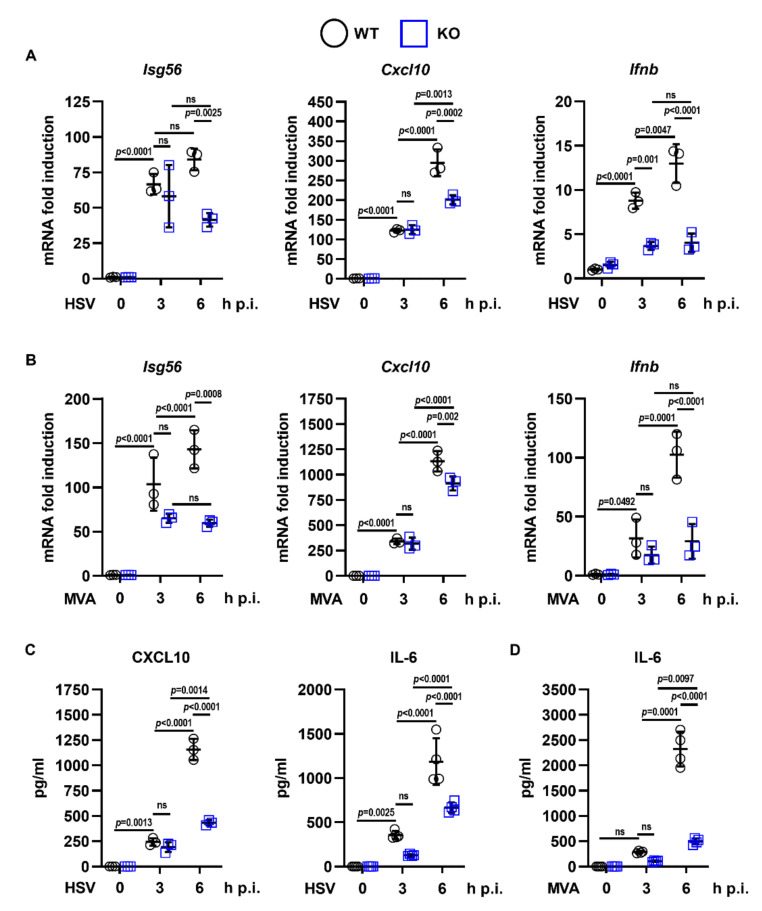
DDX50 is required for IRF3-dependent signalling in response to the dsDNA viruses HSV-1 and VACV. WT MEFs or *Ddx50^−/−^* MEFs were infected for 3 or 6 h at 10 p.f.u./cell with HSV-1 S17 ΔICP0 (**A**,**C**) or MVA (**B**,**D**) or left uninfected. (**A**,**B**) mRNA was extracted and *Ifnb*, *Isg56*, and *Cxcl10* levels were analysed by RT-qPCR relative to *Gapdh*. Representative of at least two independent experiments. (**C**,**D**) Secretion of CXCL10 and IL-6 were measured at 3 and 6 h post infection by ELISA. Representative of three independent experiments performed in quadruplicate. For all panels, statistical significance was determined by performing a two-way ANOVA test followed by Tukey’s multiple comparison post-hoc test. ns, non-significant.

Importantly, the defect in NF-κB/IRF3-dependent gene expression in response to PolyIC was rescued by transduction and complementation of *Ddx50* KO cells with a lentiviral vector encoding *Ddx50* (Figure 2A–C). This ruled out CRISPR off-target effects for the observed defect. Furthermore, overexpression of DDX50 augmented *IFNβ* promoter activity (Figure S2A,B) and secretion of CXCL10 and IL-6 in response to PolyIC transfection (Figure S2C,D). Interestingly, overexpression of DDX50 alone resulted in increased expression and secretion of CXCL10 (Figure 2A,B and Figure S2C), indicating pathway activation above basal level even in the absence of stimulation. This may be due to the higher expression of DDX50 in the complemented cell line compared to the WT endogenous levels. In non-stimulated cells, overexpression may lead to autoactivation of IRF3/NF-κB-dependent gene expression, and cytokine synthesis, resulting in higher levels of CXCL10. To investigate whether loss of DDX50 affected signalling in another mammalian species, we knocked out DDX50 from human embryonic kidney (HEK293T) cells and found that in HEK293T *DDX50* KO lines, there was a defect in pathway activation in response to SeV infection (Figure 1E,F and Figure S1). To investigate the biological relevance of DDX50, WT or KO MEFs were infected with two large dsDNA viruses: VACV and HSV-1. The modified vaccinia Ankara (MVA) strain and the HSV-1 ΔICP0 strain each elicit strong innate immune responses in tissue culture and were therefore used to increase pathway activation and sensitivity, as described previously [30,38]. Following infection with either MVA or HSV-1 ΔICP0, the expression of IRF3 and/or NF-κB-dependent *Isg56*, *Cxcl10*, and *Ifnb* were significantly upregulated in WT cells and showed expected kinetics with significantly higher expression at 6 h p.i. in comparison to non-stimulated control cells and 3 h p.i. (Figure 3A,B). Significantly, in the KO cells, expression was diminished in comparison to WT cells with the defect more pronounced at 6 h p.i. in comparison to 3 h p.i. (Figure 3A,B). This correlated with the kinetics and decreased secretion of CXCL10 and IL-6 as determined by ELISA (Figure 3C,D). Following infection with either virus, the effect of *Ddx50* KO on *Cxcl10* expression, although significant, was less pronounced in comparison to *Isg56* and *Ifnb* (Figure 3A,B). Overall, this highlights the importance of DDX50 in innate immune signalling during viral infection.

### 3.2. Loss of Ddx50 Does Not Alter IL-1α or TNFα-Mediated NF-κB Activation

Deletion of *Ddx50* impaired the induction of NF-κB/IRF3-dependent genes in response to dsRNA transfection, ssRNA virus infection (Figure 1), and dsDNA virus infection (Figure 3), and previously was reported to modulate MAP kinase signalling [26]. Therefore, alternative pathways were tested to determine if the observed defect was specific to RNA-dependent signalling. WT or KO MEFs were treated with IL-1α or TNFα and activation of the NF-κB promoter or expression of *Il-6* and *Nfkbia* were measured by Luciferase reporter gene assay or RT-qPCR, respectively. No differences in NF-κB promoter activity or NF-κB-dependent gene expression were observed (Figure S3A–C), indicating that DDX50 does not play a role in canonical IL-1 receptor- or TNF receptor-induced NF-κB signalling. Together, these data suggest DDX50 acts at the stage of IRF3 activation specifically, at or upstream of MAVS and/or TRIF activation before the RNA sensing pathways diverge to activate IRF3 and NF-κB.

### 3.3. DDX50 Accumulates in the Cytoplasm to Activate Signalling Upstream of MAVS

**Figure 4:**
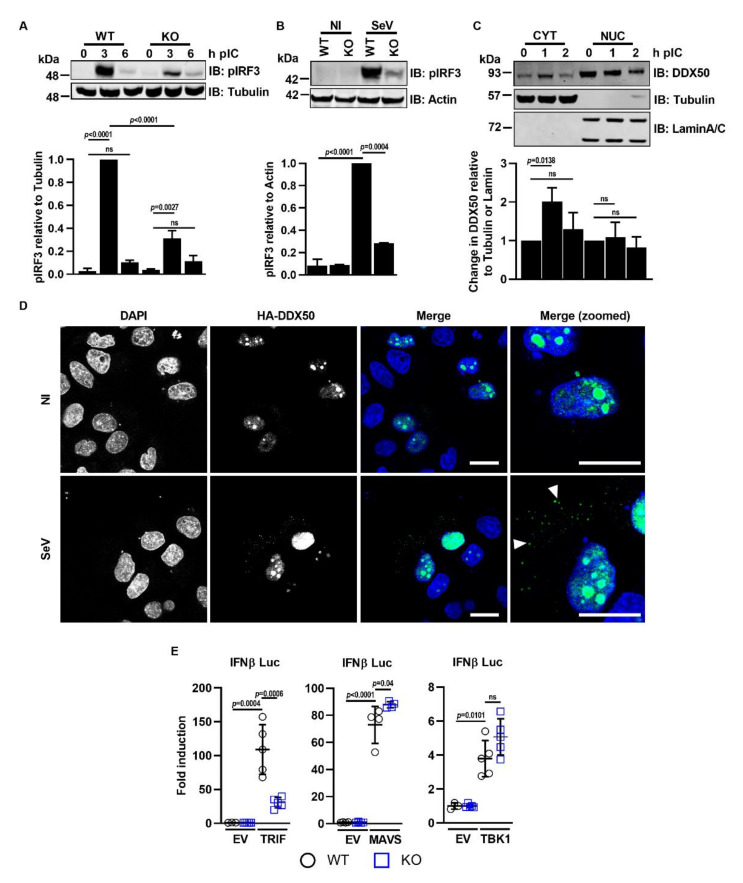
DDX50 accumulates in the cytoplasm in response to cytoplasmic dsRNA and acts upstream or independently of MAVS activation. (**A**,**B**) Representative immunoblot of phosphorylated IRF3 at (**A**) Ser396 or (**B**) Ser386 (pIRF3) for (**A**) WT or *Ddx50^−/−^* MEFs transfected with lipofectamine only or 5 µg/mL PolyIC for 3 and 6 h or (**B**) WT and DDX50^−/−^ HEK293Ts untreated or infected with SeV for 18 h. The level of IRF3 phosphorylation was calculated by densitometry, relative to α-tubulin (**A**) or actin (**B**) and is representative of at least two independent experiments. (**C**) Representative immunoblot following transfection of WT MEFs with 2.5 µg/mL PolyIC for the indicated times and isolation of the cytoplasmic (cyt) and nuclear fractions (nuc). Immunoblots were stained for DDX50 or α-tubulin and lamin A/C as cytoplasmic and nuclear fraction controls, respectively. The change in the level of cytoplasmic and nuclear DDX50 was calculated by densitometry, relative to the DDX50 levels at time point 0 in the cytoplasmic or nuclear fractions and to the α-tubulin or Lamin A/C levels, respectively. Representative of three independent experiments. Statistical significance was determined by performing a one-way ANOVA test followed by Tukey’s multiple comparison post-hoc test. (**D**) Immunofluorescence staining for DDX50 localisation. HeLa cells were transfected with pLDT-DDX50-HA and left uninfected (NI) or infected for 1.5 h with SeV at 40 HAU/mL. DDX50 localisation was visualised using an anti-HA antibody. Puntca are indicated with white arrows. DAPI was used to stain the nucleus. Representative of three independent experiments. Scale bar, 10 µM. (**E**) Luciferase activity of WT or *Ddx50^−/−^* MEFs co-transfected with EV or indicated plasmids along with plasmids encoding Firefly Luciferase under the *Ifnβ* promoter and Renilla as an internal control. Experiments shown are representative of at least three independent experiments. For all panels, unless stated otherwise, statistical significance was determined by performing a two-way ANOVA test followed by Tukey’s multiple comparison post-hoc test.

To investigate where DDX50 acts in the pathway and determine how it facilitates activation of IRF3/NF-κB in response to dsRNA, the phosphorylation of IRF3 was examined. This is a key step in IRF3-dependent signalling and leads to IRF3 dimerisation, translocation into the nucleus, and IRF3-dependent gene transcription. Following its phosphorylation and activation, IRF3 signalling is tightly regulated and is controlled by a negative feedback loop leading to its ubiquitination and degradation [39]. Upon PolyIC transfection of MEFs or SeV infection of HEK293Ts, the activation of IRF3 in WT cells followed the expected pattern and kinetics of Ser386/396 phosphorylation (Figure 4A,B). However, IRF3 phosphorylation was diminished in DDX50 KO cells (Figure 4A,B), mapping DDX50 function upstream of IRF3 phosphorylation.

DDX50 is reported to reside in the nucleolus. However, to act upstream of IRF3 phosphorylation in the canonical cascade, one would expect DDX50 to be cytosolic. To explore this further, biochemical fractionation of MEFs and anti-DDX50 immunoblotting with or without prior pathway stimulation was used to assess the subcellular localisation of DDX50 at different times after the addition of cytoplasmic dsRNA. LaminA/C and α-tubulin served as nuclear and cytoplasmic fraction controls, respectively. As described, under resting conditions, the majority of DDX50 was in the nuclear fraction [25] (Figure 4C). However, DDX50 accumulated in the cytoplasm 1 h post-stimulation (Figure 4C). At 2 h post-stimulation, the level of DDX50 in the cytoplasm returned to basal levels (Figure 4C). To support this finding, the assay was repeated and the localisation of HA-tagged DDX50 was analysed by immunofluorescence. Under resting conditions, DDX50 was restricted to the nucleolus, with weak nuclear staining. In agreement with the biochemical fractionation assay, accumulation of DDX50 in distinct cytoplasmic puncta was observed 1 h post infection with SeV (Figure 4D). The nucleocytoplasmic shuttling of DDX50 upon stimulation led us to investigate at which stage in the activation of the IRF3/NF-κB pathway DDX50 might function. The IRF3/NF-κB pathway can be activated by transfection and overexpression of key proteins acting at specific stages of the pathway. Therefore, to map in more detail where DDX50 acts, plasmids encoding TBK1, MAVS, or TRIF were co-transfected into WT or KO MEFs along with pLuc-IFNβ and pTK-RL. Activation of the pathway was measured by Firefly and Renilla Luciferase activation as before. No differences in fold activation were observed between the WT and KO cells upon expression of TBK1 or MAVS (Figure 4E). However, activation was significantly impaired in the *Ddx50* KO cell line upon expression of TRIF, mapping DDX50 upstream or independently of MAVS, but at or downstream of TRIF activation. Notably, DDX50 shares 55.6% amino acid identity with DDX21, which is essential for TRIF recruitment via complex formation with DDX1 and DHX36 in response to cytoplasmic dsRNA [17].

### 3.4. DDX50 Co-Immunoprecipitates with TRIF and Activates Signal Transduction Independently of the DDX1-DDX21-DHX36 Complex

**Figure 5:**
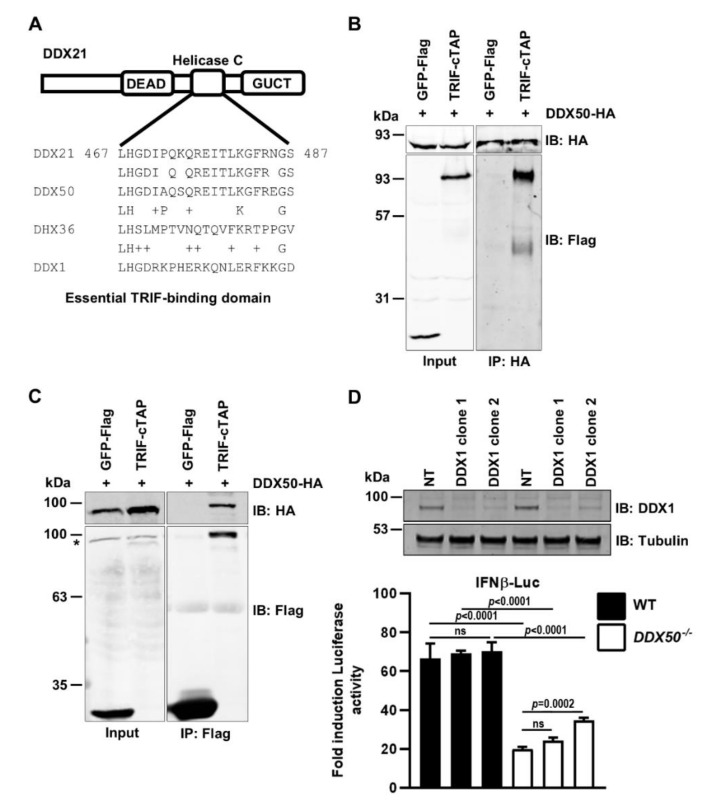
DDX50 co-IPs TRIF but acts independently of the DDX1 complex (**A**) Schematic depicting DDX21 and the corresponding region in DDX50 or DDX1 and DHX36 as a comparison. (**B**) Immunoblots from co-IP experiments of MEF DDX50-HA cell lines transiently transfected with GFP-Flag or TRIF-cTAP 1 h post-stimulation with 5 µg/mL PolyIC. (**C**) Immunoblots from co-IP experiments of HeLa cell lines transiently transfected with DDX50-HA along with GFP-Flag or TRIF-cTAP 1 h post-stimulation with 5 µg/mL PolyIC. Representative of two independent experiments. *, non-specific band. (**D**) WT or DDX50 KO HEK293T were transduced with non-targeting (NT) shRNA or with two independent clones of shRNA targeting DDX1. Knockdown was verified by immunoblotting for DDX1 and alpha-tubulin. Firefly luciferase activity of the indicated knockdown cell lines following transfection with plasmids encoding Firefly Luciferase under the *Ifnβ* promoter and Renilla. Cells were left uninfected or infected with Sendai virus (SeV) for 24 h. Representative of 3 independent experiments. Significance was determined by performing the two-way ANOVA test followed by Tukey’s multiple comparison post-hoc test. IB, immunoblot; IP, immunoprecipitation; ns, non-significant.

An essential TRIF-binding domain of DDX21 was mapped to residues 467–487 within the RNA helicase C domain [17]. Strikingly, this motif shares 86% amino acid identity with DDX50 (Figure 5A). This level of sequence conservation was specific for DDX50 and not due to the helicase C domain consensus sequence, because it was not detected within other DExD/H-box family members, such as DHX36 (Figure 5A). Due to the high level of aa identity between DDX21 and DDX50 within this region we investigated whether DDX50 can co-immunoprecipitate TRIF. Given little DDX50 is detected in the cytoplasmic fraction in the absence of stimulation, co-immunoprecipitation assays were performed using extracts of stimulated MEF cell lines that stably expressed DDX50-HA and that were transfected with TRIF-cTAP or GFP-Flag. Following stimulation, DDX50-HA specifically co-immunoprecipitated TRIF-cTAP (Figure 5B). This was confirmed by reciprocal immunoprecipitation in HeLa cells, where hDDX50-HA specifically co-immunoprecipitated with TRIF-cTAP (Figure 5C). Due to the quality of available anti-TRIF antibodies, co-immunoprecipitation of endogenous TRIF could not be tested. Furthermore, whether interaction is induced in response to stimulation or is constitutive could not be investigated due to the necessity of using stimulated cells. This led to the hypothesis that DDX50 may function with the known DDX1-DDX21-DHX36 cytoplasmic RNA sensing complex to activate TRIF-dependent NF-κB and IRF3 activation. To determine if DDX50 acts in concert with or independently of DDX1 signalling, DDX1 was depleted in WT and KO HEK293T cells and *IFNβ* promoter activity in response to SeV infection was investigated. DDX1 was efficiently (>85%) knocked down with two independent shRNAs in both the WT and DDX50 KO HEK293Ts (Figure 5D). Interestingly, DDX1 was dispensable for promoter activation in response to SeV, with no significant difference in activation compared to the non-targeting control shRNA in both the WT and KO background (Figure 5D). This suggests that DDX50 can act independently of the described DDX1-dependent complex.

### 3.5. DDX50 Is a Viral Restriction Factor

**Figure 6:**
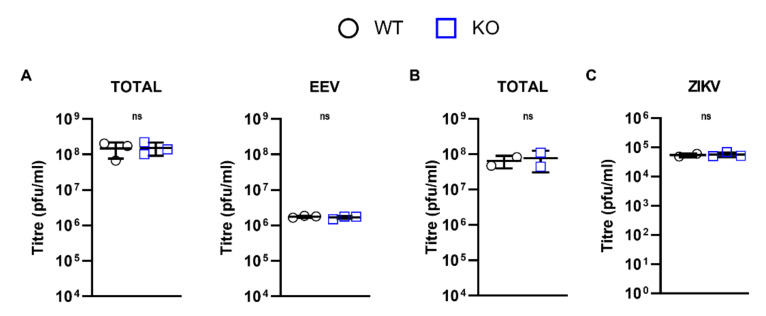
DDX50 does not impact viral replication after high MOI. (**A**) WT or *Ddx50^−/−^* MEFs were infected with vA5-GFP at 5 p.f.u./cell for 24 h. Viral titres were determined by plaque assay of the infectvity within the medium only (EEV) or total (medium plus cells) on BS-C-1 cells. Average of three independent experiments. (**B**,**C**) WT or *Ddx50^−/−^* HEK293Ts were infected with (**B**) vA5-GFP at 5 p.f.u./cell for 16 h or (**C**) ZIKV at 1 p.f.u./cell for 72 h. Viral titres were calculated by titration of cell lysates on Vero E6. Average of two independent experiments. For all panels, statistical significance was determined by performing a two-tailed unpaired *t*-test. Ns, non-significant.

IRF3 is a crucial viral restriction factor that controls the transcriptional upregulation of cytokines, chemokines, viral restriction factors, and type I IFNs and thereafter IFN-stimulated genes (ISGs) downstream of IFN-induced signalling. Given the role of DDX50 in IRF3-dependent signalling, its potential as a viral restriction factor was investigated. WT and DDX50 KO cells were infected at either high MOI or low MOI with the dsDNA viruses VACV (MEF, 5 or 0.0001 p.f.u./cell; HEK293T, 5 or 0.0003 p.f.u./cell) and HSV-1 (0.01 p.f.u./cell) or ZIKV (1 or 0.1 p.f.u./cell), an ssRNA virus, and virus replication and dissemination were analysed by virus titration and plaque formation. VACV infection produces both single-enveloped intracellular mature virus (IMV) and double-enveloped cell-associated enveloped virus (CEV) and extracellular enveloped virus (EEV) [40]. CEVs induce the formation of actin tails to propel virions towards uninfected neighbouring cells. Alternatively, EEVs are released from infected cells and mediate long-range dissemination [41]. To investigate if loss of DDX50 alters viral replication or release, VACV strain Western Reserve (WR) encoding GFP fused to the virus capsid protein A5 (A5-GFP VACV) was used to infect WT or KO MEFs/HEK293Ts at 5 p.f.u./cell and the total virus or extracellular virus titres 24 h p.i. were determined by plaque assay. No differences in the titres of cell-associated virus (IMV plus CEV) or released virus (EEV) were observed (Figure 6A,B) and equal amounts of EEV were produced (approximately 2% of the total titre; Figure 6A). Given that the activation of IRF3 restricts RNA virus infection as well, a ZIKV replication assay was performed in the absence of DDX50. Parental HEK293T and derived DDX50^−/−^ cells were infected with ZIKV at 1 p.f.u./cell. Three days p.i., supernatants of infected cells were collected, and infectious virus was titrated by plaque assay on Vero E6 cells. In concordance with dsDNA viral infection, no difference was observed at high MOI (Figure 6C).

**Figure 7:**
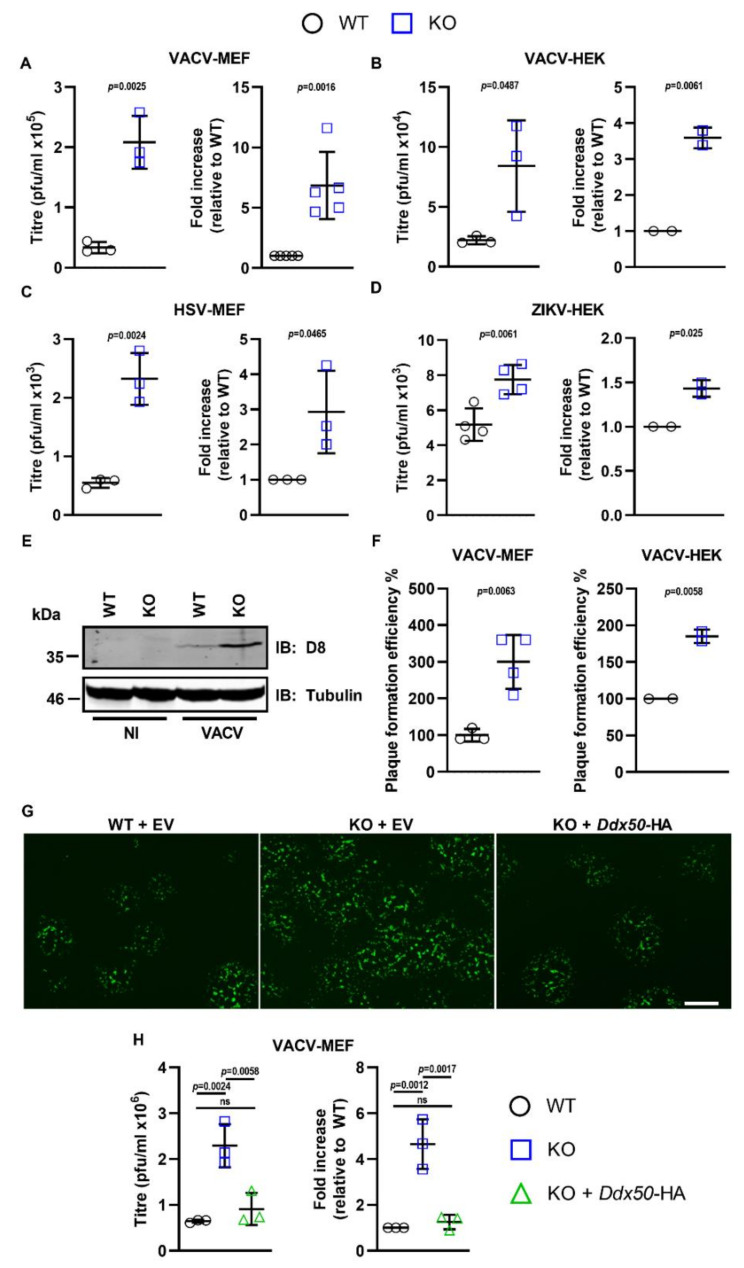
DDX50 is a viral restriction factor. (**A**,**B**) Monolayers of WT or *Ddx50^−/−^* MEFs were infected with vA5-GFP at 0.0001 p.f.u./cell. Viral titres at 24 h p.i. were determined by plaque assay on BS-C-1 cells and are represented as p.f.u./mL (left panel) or fold increase in replication relative to WT cells (right panel). (**B**) As in (**A**) but using WT or *DDX50^−/−^* HEK293Ts infected at 0.0003 p.f.u./cell for 48 h. Results shown are representative of 2 independent experiments. (**C**) Monolayers of WT or *Ddx50^−/−^* MEFs were infected with HSV-1 S17 Vp26-GFP at 0.01 p.f.u./cell. Viral titres at 48 h p.i. were determined by plaque assay on U2OS cells and are represented as p.f.u./mL (left panel) and fold increase in replication relative to WT cells (right panel). (**D**) WT or *DDX50^−/−^* HEK293Ts were infected with ZIKV at 0.1 p.f.u./cell for 72 h. Titres were determined by plaque assay on Vero E6 cells and data are shown as for (**A**–**C**). Titres shown are an average of 2 independent experiments. (**E**) WT or *Ddx50^−/−^* MEFs were infected with vA5-GFP at 0.0001 p.f.u./cell and expression of the VACV late protein D8 was analysed by immunoblot at 24 h p.i. (**F**) Cells were infected as in (**A**,**B**) and the number of plaques formed on WT or KO cells were enumerated 24 h p.i. Data are expressed as the plaque formation efficiency on KO cells compared to WT cells. Representative of two independent experiments**.** Representative of two independent experiments. (**G**,**H**) WT or *Ddx50^−/−^* MEFs transduced with pCW57-EV or pCW57-*Ddx50*-HA were infected with vA5-GFP at 0.0001 p.f.u./cell and analysed at 24 h p.i. (**G**) Representative fluorescence images of plaques following infection. Scale bar, 500 µM. (**H**) Viral titres and fold replication at 24 h p.i. Statistical significance was determined by performing a one-way ANOVA test followed by Tukey’s multiple comparison post-hoc test. For all experiments, unless stated otherwise, titres shown are representative of at least 3 independent experiments and fold changes shown are an average of at least 2 independent experiments. For all panels, unless stated otherwise, statistical significance was determined by performing a two-tailed unpaired *t*-test.

Differences in virus replication or spread are sometimes not discernible following high MOI and therefore, virus dissemination and replication were also assessed at low MOI. Monolayers of WT or KO MEFs/HEK293Ts were infected with A5-GFP-VACV at 0.0001 or 0.0003 p.f.u./cell, with HSV-1 strain 17 (S17) encoding GFP fused to Vp26 (Vp26-GFP) at 0.01 p.f.u./cell, or with ZIKV at 0.1 p.f.u./cell and viral titres were determined. Loss of DDX50 in MEFs and HEKs conferred an approximate 6- and 3.5-fold increase in the yield of VACV at 24 and 48 h p.i., respectively (Figure 7A,B). This difference was not restricted to VACV, and loss of DDX50 resulted in an increase in the yield of HSV-1 and ZIKV following low MOI (Figure 7C,D). In line with the higher viral titres, synthesis of the VACV-specific late gene product D8 was enhanced in KO MEFs (Figure 7E).

To determine if loss of DDX50 affected plaque size or infection efficiency, the size and number of plaques formed on WT or KO cells were enumerated by fluorescence microscopy following VACV infection. Whilst no difference in plaque size was observed, notably, the number of plaques formed by VACV was increased on the KO MEFs and HEK293Ts compared to control WT cells (Figure 7F and Figure S4A). Consistent with this observation, complementation of KO MEFs with pCW57-*Ddx50*-HA but not the empty vector (EV) reduced the plaque number to WT levels (Figure 7G), as shown by representative fluorescence images, and viral yields and plaque formation efficiency (Figure 7H). Furthermore, overexpression of hDDX50-HA but not hDDX28-HA in WT human fibroblasts restricted VACV, resulting in significantly lower viral titres (Figure S4B). This suggests that DDX50 restricts plaque formation when cells are infected at low MOI and without DDX50, a greater proportion of virus particles entering cells escape host defences and establish a plaque.

Together, these results provide evidence that DDX50 promotes antiviral signalling during infection and is a restriction factor for both DNA and RNA viruses, with its loss resulting in increased viral spread and subsequent replication in tissue culture.

## 4. Discussion

Type I IFNs are critical regulators of antiviral immunity and infection control and therefore, understanding the mechanisms leading to their production during infection is important. During the last decade, much research has studied the canonical RLRs and RNA sensors RIG-I, MDA5, and TLR3, and has investigated their activation, expression, and mechanisms of regulation in response to agonists and virus infection. Zhang and colleagues described a TLR3, RIG-I, and MDA5-independent pathway in mouse dendritic cells in which cytoplasmic RNA was sensed by a complex consisting of DDX1-DDX21-DHX36, leading to recruitment of TRIF [17]. Here, DDX50 is described as a new component of the IRF3 signalling pathway. DDX50 is an RNA helicase that co-precipitates with TRIF and is an integral component for IRF3/NF-κB activation in fibroblast and epithelial cells, acting independently of the DDX1 complex. Aside from the initial in vitro characterisation of the RNA helicase functional domains of DDX50, little is understood about its cellular role. A previous study concluded that DDX50 is required for MAP kinase activation through c-Jun binding [26]. However, whilst a defect in RNA sensing and signalling was observed here, no differences in TNFR/IL-1R-dependent NF-κB signalling were detected. Differences in the signalling cascade that were observed are independent of MAP kinase and activator protein 1 (AP-1) activation, indicating that this is an independent role for DDX50.

This study found that DDX50 was required for optimal IRF3/NF-κB-dependent gene expression, and cytokine synthesis and secretion following stimulation with dsRNA, SeV infection, or infection with the dsDNA viruses HSV-1 and VACV. Further investigation found that without DDX50, IRF3 phosphorylation was impaired downstream of these stimuli but that signalling was intact following activation via MAVS overexpression. This mapped the activity of DDX50, a nucleolar protein, to early in the signalling cascade upstream or independently of MAVS activation. DDX50 shuttling and cytoplasmic accumulation in distinct puncta upon stimulation is reminiscent of DDX1/TRIF staining in response to PolyIC treatment and is consistent with a role for DDX50 in cytoplasmic shuttling and regulation of IRF3 signalling [17]. Future studies and further mechanistic characterisation of DDX50 would benefit from (i) determining whether these DDX50 puncta contain TRIF and/or MAVS, and (ii) whether TRIF binding is essential for DDX50 function. Unfortunately, attempts to identify TRIF co-localisation were not successful due to the low quality of antibodies that recognise endogenous TRIF. Moreover, whilst beyond the scope of this initial study, mutation of the DDX50 domain, predicted to mediate association with TRIF and disruption of TRIF association, may inform further on whether the antiviral activity of DDX50 is TRIF dependent. The RNA sensing complex consisting of DDX1, DHX36, and DDX21 identified by Zhang and colleagues did not report on DDX50. Its absence may be due to the fact that DDX50 was below the level of detection in the initial screen, that it plays a more significant role in non-haematopoietic cells, or that it acts independently of the DDX1 complex. In support of DDX50′s mechanistic independence from the DDX1 complex, we found DDX1 to be dispensable for pathway activation in human kidney epithelial cells. However, given DDX50 is essential for DDX21 helicase activity in vitro [25], it is possible that DDX50 may function to support DDX21 activity and our data do not rule out DDX50 associating with DDX21 or DHX36 independently of the DDX1 complex. Even though DDX50 was required for optimal signal transduction in response to agonists for RIG-I, MDA5, and TLR3, its absence did not abolish signalling in response to viral infection or stimulation. Given that DDX50 binds TRIF, a protein that is non-essential for RIG-I/MDA5 signalling, it may act independently of the RLRs for optimal antiviral signalling and restriction. Although reported to co-immunoprecipitate with the positive-sense RNA virus Dengue (DENV) RNA, whether DDX50 can act as an RNA sensor directly or functions downstream of viral RNA binding and sensing remains to be investigated [42].

Consistent with a role for DDX50 in innate immune signalling, DDX50 is shown to be a viral restriction factor. Loss of DDX50 resulted in an attenuated immune response to infection with VACV or HSV-1 and enhanced replication of VACV, ZIKV, and HSV-1 in tissue culture after low MOI infection. Low MOI allows for the infection of cells with a single virion and subsequent rounds of replication, spread, and plaque formation in the presence of an altered host response. Alternatively, high MOI is designed to achieve 100% infection of all cells, usually resulting in multiple infections of the same cell. The high MOI does not allow for spread and therefore high and low MOIs are regularly used to differentiate between mechanisms that affect viral replication directly or those that affect dissemination, spread, and infection. Notably, at low MOI, a greater number of VACV plaques were formed on KO cell lines, suggesting that DDX50 acts to restrict viral infection and in its absence a greater proportion of infecting virus particles escape host defences and lead to plaque formation. In support of this, at high MOI, there were no differences in virus yield, suggesting that infection of a single cell by many incoming virus particles can overcome DDX50-mediated restriction. This is reminiscent of cellular restriction factors involved in innate immune signalling. These data provide evidence of the biological relevance of DDX50 during infection, with the increased plaque formation efficiency on DDX50 KO cells correlating with an early defect in IRF3-dependent antiviral signalling, although we cannot rule out alternative mechanisms contributing to DDX50-dependent restriction, with many DExD/H-Box RNA helicases reported to restrict viral replication independently of any known role in antiviral signalling [20]. Interestingly, recent publications using siRNA knockdown of DDX50 suggest that it may inhibit DENV replication [43,44]. Following knockdown, the authors reported a reduction in *IFNβ* promoter activity and therefore hypothesised that DDX50 may regulate type I IFN production during DENV infection [44]. This is consistent with our findings that the ZIKV titre is increased in the absence of DDX50, and together provides evidence that DDX50 is a viral restriction factor in response to multiple RNA and DNA viruses. Therefore, DDX50 as a restriction factor may extend beyond the viruses tested in this study and act broadly to activate IRF3-dependent gene transcription and restrict viral replication.

Whilst this study identifies a role for DDX50 in signalling following RNA sensing, both HSV-1 and VACV are DNA viruses. HSV-1 is reported to be restricted mostly by the cGAS-STING pathway [45]. However, DNA sensing and antiviral signalling is positively regulated by both TRIF and RNA sensing during HSV-1 infection including host RNAs [46,47,48], highlighting the essential role of RNA sensors during DNA virus infection. Cells infected with VACV contain large amounts of dsRNA late during infection [49,50]. This is due to the virus’ intermediate and late genes lacking specific transcriptional termination sequences and so lengthy overlapping transcripts are produced that hybridise to form dsRNA [51]. These transcripts can be sensed and activate innate immune signalling pathways [52]. In addition, such dsRNA can bind to and activate IFN-induced proteins, such as PKR and 2′-5′ oligoadenylate synthetase (OAS), to mediate translational shutoff. The importance of dsRNA in activating host defences is illustrated by the fact that VACV, despite being a dsDNA virus, encodes a dsRNA binding protein called E3 [53], which contributes to virulence [54]. It is important to note that TRIF is also an essential component of the STING pathway [46]. Therefore, the level to which DDX50 restricts DNA viruses in an RNA-sensing dependent manner, or whether it can further influence TRIF signalling in the cGAS-STING pathway, warrants future investigation. Furthermore, due to several innate immune sensors that were characterised in vitro failing to show a significant role in animal models, the importance of DDX50 in RNA sensing and its contribution in antiviral immunity requires validation in vivo. Unfortunately, to date, there are no KO mice or models available; however, with the recent success in generating *Ddx21* KO mice, it may soon be a plausible avenue for investigation.

## 5. Conclusions

In conclusion, this study identified DExD-Box RNA helicase DDX50 as a crucial component facilitating DDX1-independent IRF3 activation following stimulation with dsRNA or viral infection and further established a pivotal role for DDX50 as a viral restriction factor for RNA and DNA viruses.

## Data Availability

The datasets generated for this study can be found at Figshare, https://doi.org/10.6084/m9.figshare.14182832, accessed on 1 February 2022.

## Supplementary Materials

The following are available online at https://www.mdpi.com/article/10.3390/v14020316/s1, Figure S1: CRISPR-Cas9 mediated knockout of *Ddx50/DDX50* (*RH-II/Guβ*), Figure S2: DDX50 overexpression augments the innate immune response to nucleic acid, Figure S3: DDX50 (RH-II/Guβ) is not required for NF-κB-dependent gene transcription, Figure S4: Overexpression of DDX50 inhibits VACV dissemination and replication, Table S1: Constructs and primers used in this study, Table S2: Primers for qPCR and uncropped images for immunoblots used in this study.
