# Supplementary material for: DDX50 Is a Viral Restriction Factor That Enhances IRF3 Activation
Source: Viruses. 2022 Feb 3;14(2):316. doi: 10.3390/v14020316 (PMC8875258; doi:10.3390/v14020316)
Supplement: Supplementary file 1 [file viruses-14-00316-s001.zip › viruses-1529706-supplementary.pdf]

viruses

MDPI

Supplementary materials

DDX50 Is a Viral Restriction Factor That Enhances IRF3 Activation

Table S1. Constructs and primers used in the study.

Plasmids	Description		Reference/Source
pCW57-GFP-P2A-MCS	Lentiviral expression plasmid with GFP in MSC1 and P2A skip sequence followed by MCS2 under the control of a CMV promoter, Amp^r and Puro^r		Addgene, 71783
	Primer	RE	
pCW57-GFP-P2A-*Ddx50*-HA	CGACGCGTATGCCCGGGAAACTCCTCTGG<br>GCAGGATCCTTAAGCGTAATCTGGAACATCGTAT	MluI<br>BamHI	This study
pCMV-PACK	Packaging plasmid for lentivirus production with HIV Gag, Pol, Rev and Tat under the CMV promotor, Amp^r		Gift, Dr. H. Laman
pCMV-ENV	VSV-G pseudotyped envelope protein under the CMV promoter for lentivirus production, Amp^r		Gift, Dr. H. Laman
pLDT-TetR	Lentiviral expression plasmid constitutively expressing the Tetracyline promoter repressor, Amp^r and Neo^r		(Everett et al, 2013)
pLDT-MCS	Lentiviral expression plasmid with expression under the control of a Tetracycline inducible CMV promoter, Amp^r and Puro^r		(Everett et al, 2013)
	Primer	RE	
pLDT-*DDX50*-HA	CGGCTAGCATGCCTGGGAAACTCCTCTGG<br>CGGATATCTTAAGCGTAATCTGGAACATCGTATGGGTAG-<br>TCAAAACTCCGTT<br>TGTGGCC	NheI<br>EcoRV	This study
pLDT-*DDX28*-HA	CGGATATCATGGCTCTAACGCGGCCGGT<br>CGGATATCTTAAGCGTAATCTGGAACATCGTATGGGTAGGTTGCTT-<br>GGGGCA<br>AAGGCTC	EcoRV<br>EcoRI	This study
pLDT-*Ddx50*	CGGCTAGCATGCCCGGGAAACTCCTCTGG<br>CGGATATCTCAGTCAAAATTCCGTTTATG	NheI<br>EcoRV	This study
pCDNA4/TO-nTAP	Mammalian expression plasmid with expression under the control of a Tetracycline inducible CMV promoter, Amp^r and Zeocin^r		(Pallett et al, 2019)
pMX-CMV-YFP	Retroviral plasmid for viral package signal, transcription and processing, YFP expression and puromycin selectable, Amp^r		Invitrogen
pMX-CMV-YFP-miR30E *DDX1* clone 1	tcgagaaggtatattgctgttgacagtgagcgcccgggcaatcaaggaacataatagtgaagccacagat<br>cttgaagtccgaggcagtaggcatccgggcaatcaaggaacataatacatctgtggcttcactattatgt		This study
pMX-CMV-YFP-miR30E *DDX1* clone 2	tcgagaaggtatattgctgttgacagtgagcgcgatgtggtctgaagctattaatagtgaagccacagat<br>cttgaagtccgaggcagtaggcaagatgtggtctgaagctattaatacatctgtggcttcactattaata		This study
pMX-CMV-YFP-miR30E *LacZ*	tcgagaaggtatattgctgttgacagtgagcgACGTCGTATTACAACGTCGTGAtagtgaa-<br>gccacagat<br>cttgaagtccgaggcagtaggcaCCGTCGTATTACAACGTCGTGAtacatctgtggcttcac-<br>taTCACGA		This study

Amp^r, Ampicillin resistance; Puro^r, Puromycin resistance; Neo^r, Neomycin resistance; Zeocin^r, Zeocin resistance; RE, restriction enzyme.

Table S2. Primers for qPCR.

Primer	Sequence
Ifnb Fwd	CATCAACTATAAGCAGCTCCA
Ifnb Rev	TTCAAGTGGAGAGCAGTTGAG
Il-6 Fwd	GTAGCTATGGTACTCCAGAAG
Il-6 Rev	ACGATGATGCACTTGCAGAA
Cxcl10 Fwd	ACTGCATCCATATCGATGAC
Cxcl10 Rev	TTCATCGTGGCAATGATCTC
Isg56 Fwd	ACCATGGGAGAGAATGCTGAT
Isg56 Rev	GCCAGGAGGTTGTGC
Nfkbia Fwd	CTGCAGGCCACCACCTACAA
Nfkbia Rev	CAGCACCCAAAGTCACCAAGT
Gapdh Fwd	ATCAACGACCCCTTCATTGACC
Gapdh Rev	CCAGTAGACTCCACGACATACTCAGC

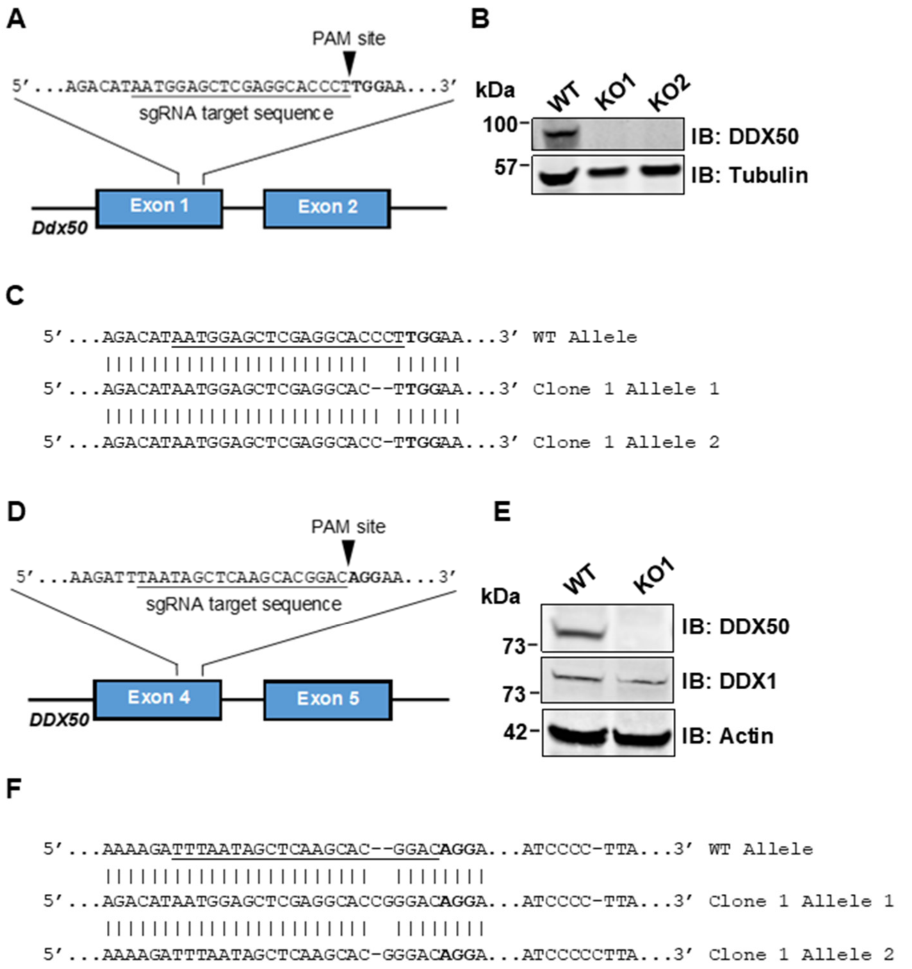

Figure S1. CRISPR-Cas9 mediated knockout of *Ddx50*/*DDX50* (*RH-II*/*Guβ*). WT mouse **(A-C)** fibroblasts and human HEK293Ts **(D-F)** were transfected with pX459 - sgRNA targeting *Ddx50* exon 1 **(A)** and *DDX50* exon 4 **(D)**, respectively. Cells were selected in 4 μg/ml (MEFs) or 1 μg/ml (HEK293T) puromycin for 2 weeks before clones were expanded. **(A** and **D)** Schematic representation of the genomic target for each mutation. **(B** and **E)** SDS-PAGE and immunoblot to assess levels of DDX50. KO, Knockout clone. **(C** and **F)** Single allele sequencing of exon 1 and exon 4 for *Ddx50* and *DDX50*, respectively.

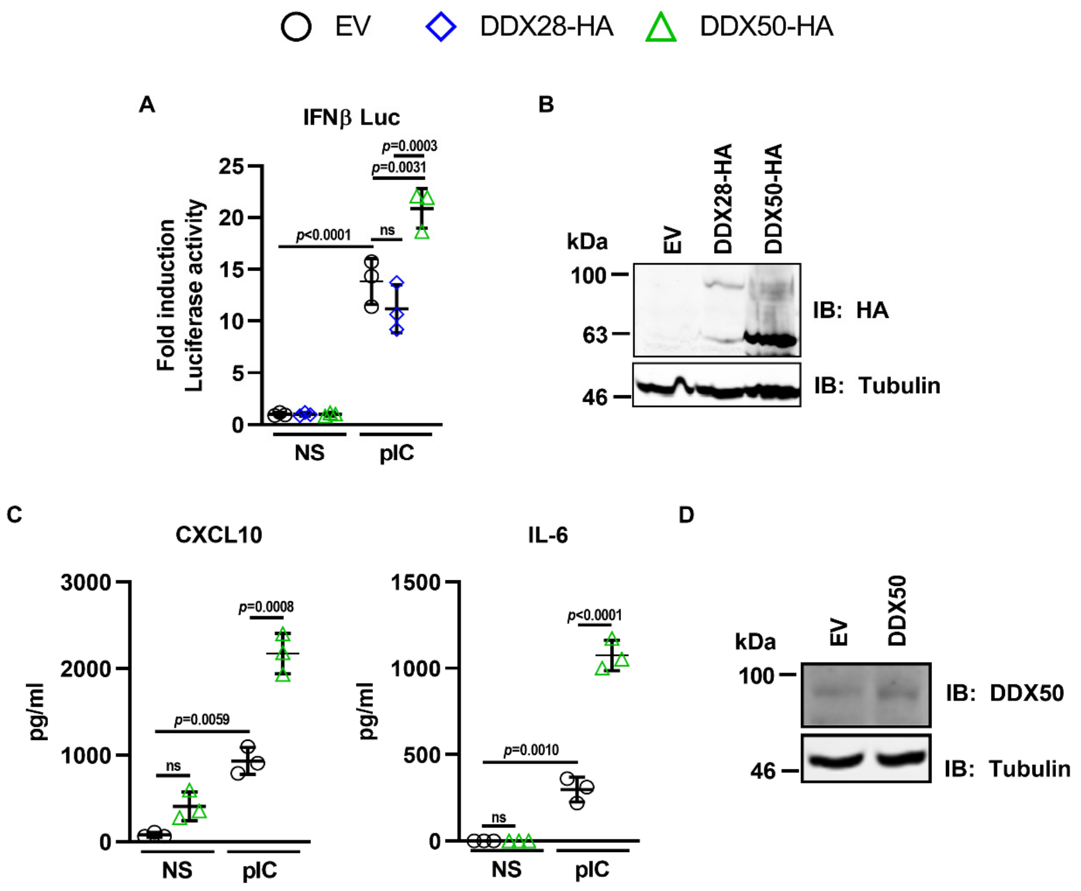

Figure S2. DDX50 overexpression augments nucleic acid sensing. **(A)** The human fibroblast (HF) EV, DDX50-HA or DDX28-HA cell lines were transfected with plasmids encoding Firefly Luciferase under the *Ifnβ* promoter or Renilla, and Firefly Luciferase fold induction was analysed following transfection with 5 μg/ml Poly IC for 6 h. **(B)** Relative levels of DDX28 and DDX50 expression were confirmed by immunoblotting with anti-HA and anti-α-tubulin. Representative of three independent experiments. **(C)** WT MEF EV or DDX50 transduced cell lines were transfected with lipofectamine only or 5 μg/ml PolyIC for 7 h and the secretion of CXCL10 and IL-6 was measured by ELISA. Representative of at least two independent experiments. For all panels statistical significance was determined by performing a Two-way ANOVA test followed by Tukey's multiple comparison post-hoc test. ns, non-significant. **(D)** Overexpression of DDX50 was confirmed by immunoblotting with anti-DDX50 and anti-α-tubulin.

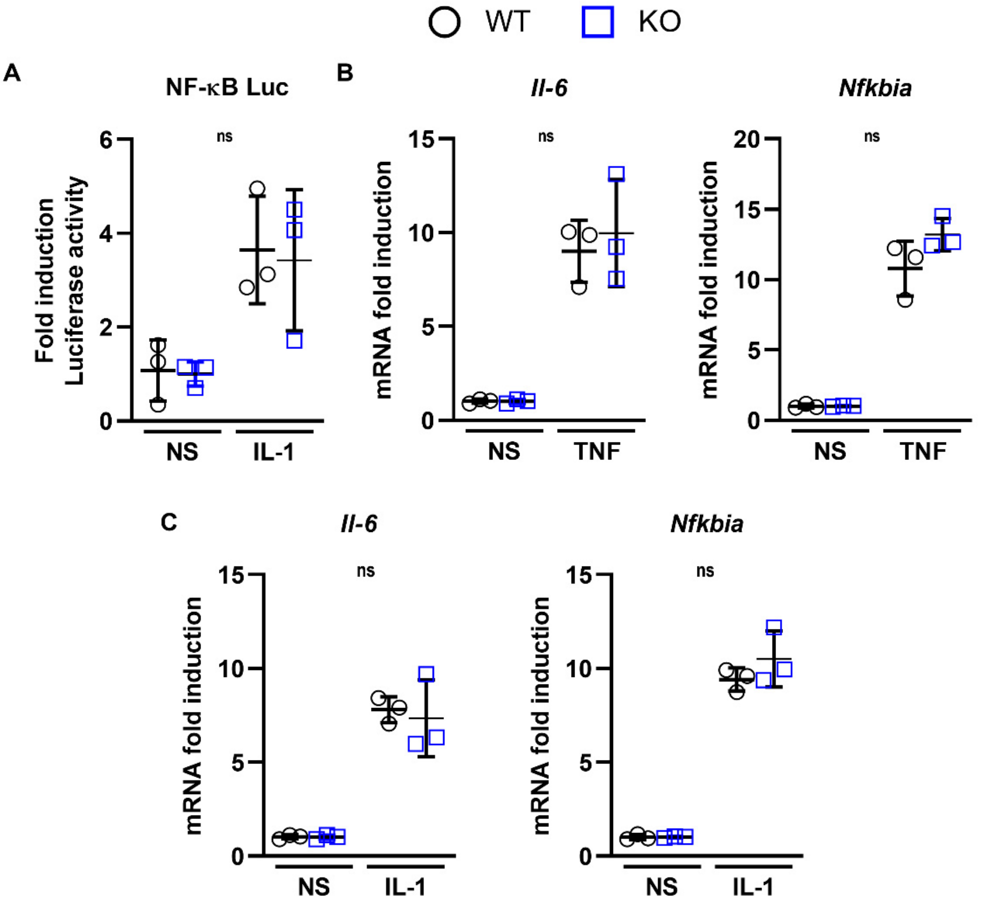

Figure S3. DDX50 (RH-II/Guβ) is not required for NF-κB-dependent gene transcription. **(A)** WT or *Ddx50*$^{-/-}$ MEFs were transfected with pNF-κB-Luc or pTK-RL, as an internal control. Cells were left untreated or stimulated for 7 h with 100 ng/ml IL-1*α* and Firefly Luciferase activity was measured. **(B** and **C)** WT or *Ddx50*$^{-/-}$ MEFs were left untreated or stimulated for 1 h with 100 ng/ml TNF*α* **(B)** or IL-1*α* **(C)**. Following mRNA extraction, the fold induction of *Nfkbia* and *Il-6* mRNA levels relative to *Gapdh* were analysed by RT-qPCR. Representative of 3 independent experiments. For all panels statistical significance was determined by performing a Two-way ANOVA test followed by Tukey's multiple comparison post-hoc test. ns, non-significant.

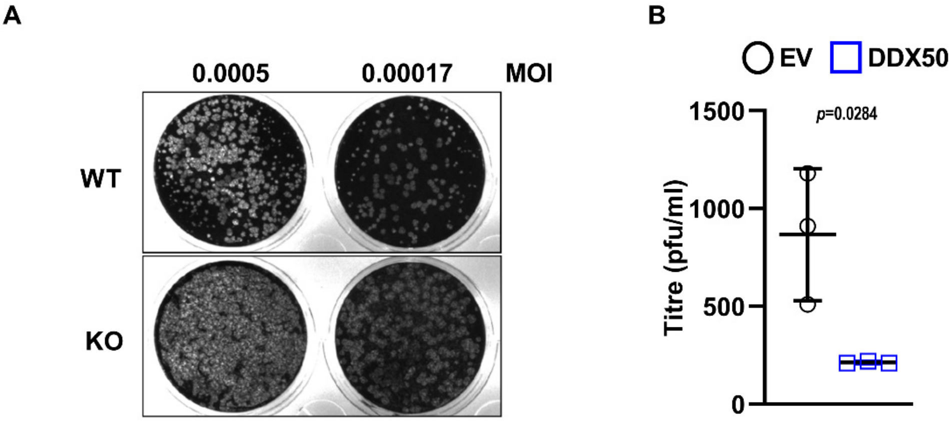

Figure S4. Overexpression of DDX50 inhibits VACV dissemination and replication. **(A)** WT or *Ddx50*$^{-/-}$ MEFs were infected with A5-GFP VACV WR at MOI = 0.0005 or 0.00017 and representative images for enumeration of plaque formation efficiency were taken 24 h p.i. **(B)** HF cells transduced with EV or to express HA tagged DDX28 or DDX50 were infected with 300 p.f.u. VACV and titre

was calculated 48 h p.i. Representative of three independent experiments. Statistical significance was determined by performing a two-tailed unpaired *t*-test.
